# Age-specific breast cancer risk by body mass index and familial risk: prospective family study cohort (ProF-SC)

**DOI:** 10.1186/s13058-018-1056-1

**Published:** 2018-11-03

**Authors:** John L. Hopper, Gillian S. Dite, Robert J. MacInnis, Yuyan Liao, Nur Zeinomar, Julia A. Knight, Melissa C. Southey, Roger L. Milne, Wendy K. Chung, Graham G. Giles, Jeanine M. Genkinger, Sue-Anne McLachlan, Michael L. Friedlander, Antonis C. Antoniou, Prue C. Weideman, Gord Glendon, Stephanie Nesci, kConFab Investigators, Irene L. Andrulis, Saundra S. Buys, Mary B. Daly, Esther M. John, Kelly Anne Phillips, Mary Beth Terry

**Affiliations:** 1 Centre for Epidemiology and Biostatistics, The University of Melbourne, Parkville, VIC Australia; 2 Cancer Epidemiology and Intelligence Division, Cancer Council Victoria, Melbourne, VIC Australia; 3 Department of Epidemiology, Mailman School of Public Health, Columbia University, 722 W 168th St, 7th Floor, New York, NY USA; 4 Lunenfeld-Tanenbaum Research Institute, Sinai Health System, Toronto, ON Canada; 5 Dalla Lana School of Public Health, University of Toronto, Toronto, ON Canada; 6 Department of Pathology, Genetic Epidemiology Laboratory, The University of Melbourne, Parkville, VIC Australia; 7 Herbert Irving Comprehensive Cancer Center, Columbia University Medical Center, New York, NY USA; 8 Departments of Pediatrics and Medicine, Columbia University, New York, NY USA; 9 Department of Medicine, St Vincent’s Hospital, The University of Melbourne, Parkville, VIC Australia; 10 Department of Medical Oncology, St Vincent’s Hospital, Fitzroy, VIC Australia; 11 Prince of Wales Clinical School, University of New South Wales, Sydney, NSW Australia; 12 Department of Medical Oncology, Prince of Wales Hospital, Randwick, NSW Australia; 13 Department of Public Health and Primary Care, Centre for Cancer Genetic Epidemiology, University of Cambridge, Cambridge, UK; 14 Division of Cancer Medicine, Peter MacCallum Cancer Centre, Melbourne, VIC Australia; 15 Sir Peter MacCallum Department of Oncology, The University of Melbourne, Melbourne, VIC Australia; 16 The Research Department, The Peter MacCallum Cancer Centre, Melbourne, VIC Australia; 17 Departments of Molecular Genetics and Laboratory Medicine and Pathobiology, University of Toronto, Toronto, ON Canada; 18 Department of Medicine and Huntsman Cancer Institute, University of Utah Health Sciences Center, Salt Lake City, UT USA; 19 Department of Clinical Genetics, Fox Chase Cancer Center, Philadelphia, PA USA; 20 Department of Medicine and Stanford Cancer Institute, Stanford University School of Medicine, Stanford, CA USA; 21 Precision Medicine, School of Clinical Sciences at Monash Health, Monash University, Clayton, CA VIC 3168 USA

**Keywords:** Breast cancer, Body mass index, Familial risk, Breast and Ovarian Analysis of Disease Incidence and Carrier Estimation Algorithm, Gene–environment interaction

## Abstract

**Background:**

The association between body mass index (BMI) and risk of breast cancer depends on time of life, but it is unknown whether this association depends on a woman’s familial risk.

**Methods:**

We conducted a prospective study of a cohort enriched for familial risk consisting of 16,035 women from 6701 families in the Breast Cancer Family Registry and the Kathleen Cunningham Foundation Consortium for Research into Familial Breast Cancer followed for up to 20 years (mean 10.5 years). There were 896 incident breast cancers (mean age at diagnosis 55.7 years). We used Cox regression to model BMI risk associations as a function of menopausal status, age, and underlying familial risk based on pedigree data using the Breast and Ovarian Analysis of Disease Incidence and Carrier Estimation Algorithm (BOADICEA), all measured at baseline.

**Results:**

The strength and direction of the BMI risk association depended on baseline menopausal status (*P* < 0.001); after adjusting for menopausal status, the association did not depend on age at baseline (*P* = 0.6). In terms of absolute risk, the negative association with BMI for premenopausal women has a much smaller influence than the positive association with BMI for postmenopausal women. Women at higher familial risk have a much larger difference in absolute risk depending on their BMI than women at lower familial risk.

**Conclusions:**

The greater a woman’s familial risk, the greater the influence of BMI on her absolute postmenopausal breast cancer risk. Given that age-adjusted BMI is correlated across adulthood, maintaining a healthy weight throughout adult life is particularly important for women with a family history of breast cancer.

## Background

Body mass index (BMI) is an intriguing risk factor for breast cancer because its association with the disease depends on time of life. Greater BMI has been found to be associated with an increased risk for postmenopausal women [1–9], while for premenopausal women, young women, and even adolescent girls [2, 4, 6, 8, 10–14], greater BMI has been found to be associated with a decreased risk. These findings have been consistent across different racial and ethnic subgroups [2, 14, 15] and across both case–control and cohort designs globally [1–4, 6–13], suggesting they are not a consequence of systematic biases [9].

BMI is an important risk factor because it is potentially modifiable. The fact that greater BMI appears to be protective at young ages, yet has the opposite association in later life, presents a potential problem for simple cancer control messaging; therefore, its consequences need to be quantified. A prospective study and a case-control study have found that the increased risk associated with higher BMI increases with time after menopause but is not evident until 10 years post menopause [16, 17].

A better understanding of how the BMI-associated risk varies with age and menopausal status is needed. It is interesting that, from genome-wide association studies, genetic risk scores based on single-nucleotide polymorphisms (SNPs) that predict higher BMI in childhood or adulthood are associated with lower risk of both premenopausal and postmenopausal breast cancer [18, 19]. Under the assumptions of Mendelian randomization, the authors concluded that these relationships were causal (even though those SNPs explained only a small proportion of the variation in BMI), thus lending additional support to the evidence that the effect of BMI varies by time of life.

Family history is another important risk factor for breast cancer that could not exist without there being a very strong gradient in underlying familial risk. To explain an overall average estimate of a 2-fold increased risk associated with having an affected first-degree relative, there must be at least a 20-fold inter-quartile risk ratio across the underlying familial causes [20]. This gives reason to consider family history not solely as a binary construct but rather as an underlying continuous measure that reflects this large gradient. Underlying familial risk can be predicted from family history using risk models that use pedigree information including age of onset of affected relatives. It is becoming increasingly possible to better differentiate women according to underlying genetic risk using SNP-based scores [21, 22]. Familial risk prediction is likely to improve with larger genome-wide association studies and the use of more informative statistical methods to create better SNP-based and family-history-based risk scores.

With the advent of gene panel testing for high-risk mutations in known breast cancer susceptibility genes [23–25], family cancer clinics will screen increasing numbers of women with a family history of breast cancer. The vast majority of these women will, however, not be found to carry a mutation that can currently be classified as deleterious. Therefore, a key clinical issue is risk management advice for women at familial risk who are found not to carry high-risk mutations. To resolve this issue, it is essential to know if their breast cancer risk factors are the same, and if the risk associations are of the same magnitude, as they are for women in the general population.

This issue of multiplicative and additive interaction with familial risk must be considered for each risk factor. If there is no difference in strength of associations by familial risk and the study is well-powered, advice on that risk factor’s relevance can be confidently given to women across the full spectrum of familial risk. In theory, if there are no interactions between risk factors on the multiplicative scale then there will be additive interactions [26, 27]. Knowledge of the extent of disease association by familial risk will enable prevention and screening measures to be appropriately offered to women.

To address the issues of whether breast cancer risk associated with BMI depends on the age of a woman, her menopausal status, and her underlying familial risk, we conducted a prospective study of women across broad ranges of age and familial risk at baseline.

## Methods

The Breast Cancer Prospective Family Study Cohort (ProF-SC) comprises baseline and follow-up data from the Breast Cancer Family Registry (BCFR) and the Kathleen Cuningham Foundation Consortium for Research into Familial Breast Cancer (kConFab) (for full details see [28]). These prospective family cohorts are enriched for familial risk of breast cancer and have accumulated up to 20 years of follow up. The BCFR is a collaboration of six breast cancer family studies from the USA, Canada and Australia, and the protocols and data collection have previously been reported for the baseline studies [29] and the follow-up studies [28]. kConFab is an Australian and New Zealand breast cancer family study, and details of the core resource [30] and follow-up study [28, 31] have been previously reported. Ethics approval for the six sites of the BCFR and for kConFab was granted by the applicable human research ethics committees at the participating institutions. All participants in the BCFR and kConFab provided written informed consent before participation.

### Recruitment and follow up

Probands and their family members were recruited to the BCFR and kConFab according to site-specific protocols [28–30, 32]. At a minimum, first-degree female relatives of the probands were recruited, and at some sites second-degree and more distant female relatives of the probands were also recruited. In the BCFR, most of the families were recruited from 1996 to 2000, with some sites recruiting new families after that time; all sites continued to recruit additional participants within these families on an ongoing basis as relatives decided to join or attained the minimum eligibility age of 18 years. Australian families recruited to an earlier study from 1992 to 1995 [33, 34] were also included, while the North American sites extended the recruitment of specific subgroups from 2001 to 2011 (minorities in Philadelphia, New York, Ontario, and Northern California; *BRCA1* and *BRCA2* mutation carriers in Utah; Ashkenazim in Ontario). For kConFab, participants were recruited continuously from 1997 onwards.

For the BCFR, systematic follow ups were conducted 10 years and 15 years after the first round of recruitment to the BCFR, while the kConFab participants have been followed up every 3 years. At follow-up, the risk factor and cancer family history questionnaires were updated and participants were asked to provide the date of death of any deceased relatives.

### Baseline questionnaires

The BCFR and kConFab used the same risk factor questionnaire [28]. At baseline, questionnaires were interviewer administered, either in person or by telephone, or administered by mail. The risk factor questionnaire asked about each participant’s demographic characteristics, height, weight, history of benign breast disease, breast and ovarian surgeries, reproductive history, and lifestyle factors. The cancer family history questionnaire asked about breast and other cancers (excluding non-melanoma skin cancer) in the participants and their first-degree and second-degree relatives. Each participant’s cancer information was obtained from one or more sources and was usually self-reported or reported by a first-degree relative. Where possible, verification of cancer diagnosis was sought through pathologist review of tissue samples, pathology reports, cancer registries, medical records, or death certificates [28–30].

### Statistical methods

We studied women who were initially unaffected by invasive breast cancer or ductal carcinoma in situ of the breast up until 3 months following completion of their baseline questionnaires. To be eligible, women also had to be aged 18 to 79 years at baseline, have at least 2 months of follow up (either by completing a questionnaire before 30 June 2011 or having a family member update their cancer and vital status), and not have had a bilateral risk-reducing mastectomy at baseline. For these analyses, we excluded 331 women for whom we did not have complete data for BMI, 1220 women for whom we were unable to determine menopausal status, and 42 women for whom we did not have complete data for both BMI and menopausal status. From the original cohort of 17,628 women, this left 16,035 (91.0%) available for analysis.

Baseline BMI was calculated as current weight (kg) divided by squared height (m) using information captured by the baseline risk factor questionnaire. We used log-transformed BMI in analyses. Baseline menopausal status was determined from questions asking about time since last menstrual period and reason for cessation of menstruation. For each participant, the 1-year risk of invasive breast cancer and the lifetime risk (risk to age 80 years from birth) were calculated using the Breast and Ovarian Analysis of Disease Incidence and Carrier Estimation Algorithm (BOADICEA) version 3 using pedigree information at baseline. This algorithm uses information on breast, ovarian, and male breast cancer and age at diagnosis for first, second, and third-degree relatives, along with date of birth, vital status, age at interview or death, and country-specific age-specific incidences [35, 36] to calculate risk. Where available, information on *BRCA1* and *BRCA2* mutation testing was also used to calculate risk. Mutations were protein-truncating or missense mutations classified as deleterious by the Breast Cancer Information Core [37]. Details of testing are given elsewhere [38]. Sensitivity of the mutation detection technique was assumed to equal 70% and 80% for *BRCA1* and *BRCA2*, respectively.

Time in the study began 2 months after the age of completion of the baseline questionnaire and ended at whichever came first of the following: age last known to be alive, diagnosis of invasive or in situ breast cancer, bilateral risk-reducing mastectomy, age 80 years, or age at death. We conducted sensitivity analyses by including only invasive breast cancers and by excluding the 652 *BRCA1* and 519 *BRCA2* mutation carriers. We also conducted sensitivity analyses by including women with missing menopausal status and including a parameter for this group.

To investigate whether the hazard ratios (HRs) for the associations between risk of breast cancer and BMI differed by the underlying familial risk, we used Cox proportional hazard models with age as the time axis and stratified by study site and birth cohort in 10-year groups. Familial risk was defined as the log 1-year incidence of breast cancer predicted by BOADICEA adjusted for age and birth cohort. We fitted interaction terms between risk factors and familial risk.

Statistical inference was made under maximum likelihood theory, including consideration of the changes in log likelihood between nested models compared with appropriate chi-squared (χ^2^) distributions (likelihood ratio criterion). We considered many reproductive and other factors (e.g. ever use of hormonal contraceptives, number of live births, ever use of hormone replacement therapy, benign breast disease, ever smoked, ever consumed alcohol, race/ethnicity, and highest education level) as potential confounders and retained only those that were nominally statistically significant. Analyses were therefore adjusted for history of benign breast disease, race/ethnicity and education. Because the cohort included families with multiple members, robust estimates of confidence intervals (CI) were calculated accounting for clustering by family. Tests of the proportional hazards assumption were based on Schoenfeld residuals. From the test for proportional hazards, we found evidence for non-proportionality only for study site. We therefore stratified all analyses by study site and there was no longer any evidence of non-proportionality. Stata version 14 [39] was used for all statistical analyses.

We plotted the predicted age-specific absolute cumulative risk for women with different BMIs and different familial risks based on BOADICEA and underlying age-specific incidences from the Surveillance, Epidemiology, and End Results Program [40–43]. We chose three scenarios of familial risk: 12% (population average), 20% and 30%, and four scenarios of BMI (20, 25, 30 and 35 kg/m^2^). All statistical tests were two sided, and *P* values < 0.05 were considered nominally statistically significant.

## Results

For the 16,035 women from 6701 families (mean 2.4 participants per family; standard deviation (SD) = 2.4; median = 2; range = 1–75), the mean age at enrollment was 47.3 years (SD = 15.4; median = 46.6; range = 18.0–79.8) and the mean duration of follow up was 10.5 years (SD = 4.7). There were 896 reported incident breast cancers (mean age at diagnosis 55.7 years, SD = 12.6). Details of the participants from the seven study sites are given in Table 1.

**Table 1:** Baseline characteristics of study cohort and unadjusted hazard ratios (HRs) and 95% confidence intervals (CIs) from Cox proportional hazards analysis

	Unaffected		Affected		HR	95% CI	*P*
	Number	Percentage	Number	Percentage			
Age at baseline, years							
18–29	2407	15.9	49	5.5	1.00	(referent)	
30–39	3087	20.4	182	20.3	1.34	0.81, 2.20	0.3
40–49	3189	21.1	231	25.8	1.18	0.65, 2.14	0.6
50–59	2869	19.0	232	25.9	1.20	0.60, 2.40	0.6
60–69	2179	14.4	166	18.5	0.98	0.46, 2.11	1.0
70–79	1408	9.3	36	4.0	0.53	0.20, 1.38	0.2
1-year BOADICEA, %							
Q1: 0–0.13	4010	26.5	84	9.4	1.00	(referent)	
Q2: 0.14–0.34	3167	23.9	188	21.0	2.14	1.51, 3.04	< 0.001
Q3: 0.35–0.53	3672	24.3	241	26.9	3.41	2.38, 4.89	< 0.001
Q4: 0.54–7.94	3840	25.4	383	42.8	5.20	3.65, 7.42	< 0.001
Body mass index, kg/m^2^							
Q1: 14.69–21.86	3811	25.2	194	21.7	1.00	(referent)	
Q2: 21.87–24.60	3766	24.9	227	25.3	1.07	0.89, 1.30	0.5
Q3: 24.61–28.56	3771	24.9	252	28.1	1.17	0.96, 1.41	0.1
Q4: 28.57–58.86	3791	25.0	223	24.9	1.05	0.87, 1.28	0.6
History of benign breast disease							
No	10,953	72.4	551	61.5	1.00	(referent)	
Yes	3878	25.6	323	36.1	1.33	1.15, 1.54	< 0.001
Menopausal status							
Premenopausal	8669	57.3	467	52.1	1.00	(referent)	
Postmenopausal	6470	42.7	429	47.9	1.02	0.81, 1.29	0.8
Race/ethnicity							
Non-Hispanic white	11,969	79.1	750	83.7	1.00	(referent)	
Black	726	4.8	29	3.2	0.60	0.40, 0.89	0.01
Hispanic	1310	8.7	52	5.8	0.74	0.53, 1.04	0.08
Asian	574	3.8	39	4.4	0.87	0.60, 1.26	0.5
Other	417	2.8	17	1.9	0.72	0.45, 1.15	0.2
Missing	143	0.9	9	1.0			
Education, highest completed							
High school or general education development	5031	33.2	260	29.0	1.00	(referent)	
Vocational, technical, or some college or university	5709	37.7	319	35.6	1.15	0.97, 1.37	0.1
Bachelor or graduate degree	4341	28.7	313	34.9	1.42	1.18, 1.70	< 0.001
Missing	58	0.4	4	0.5			

HRs are unadjusted but stratified by birth cohort (10-year groups) and study site; to account for clustering by family, robust 95% CIs are reported

*Q1–Q4* quartiles 1–4

**Fig. 1:**
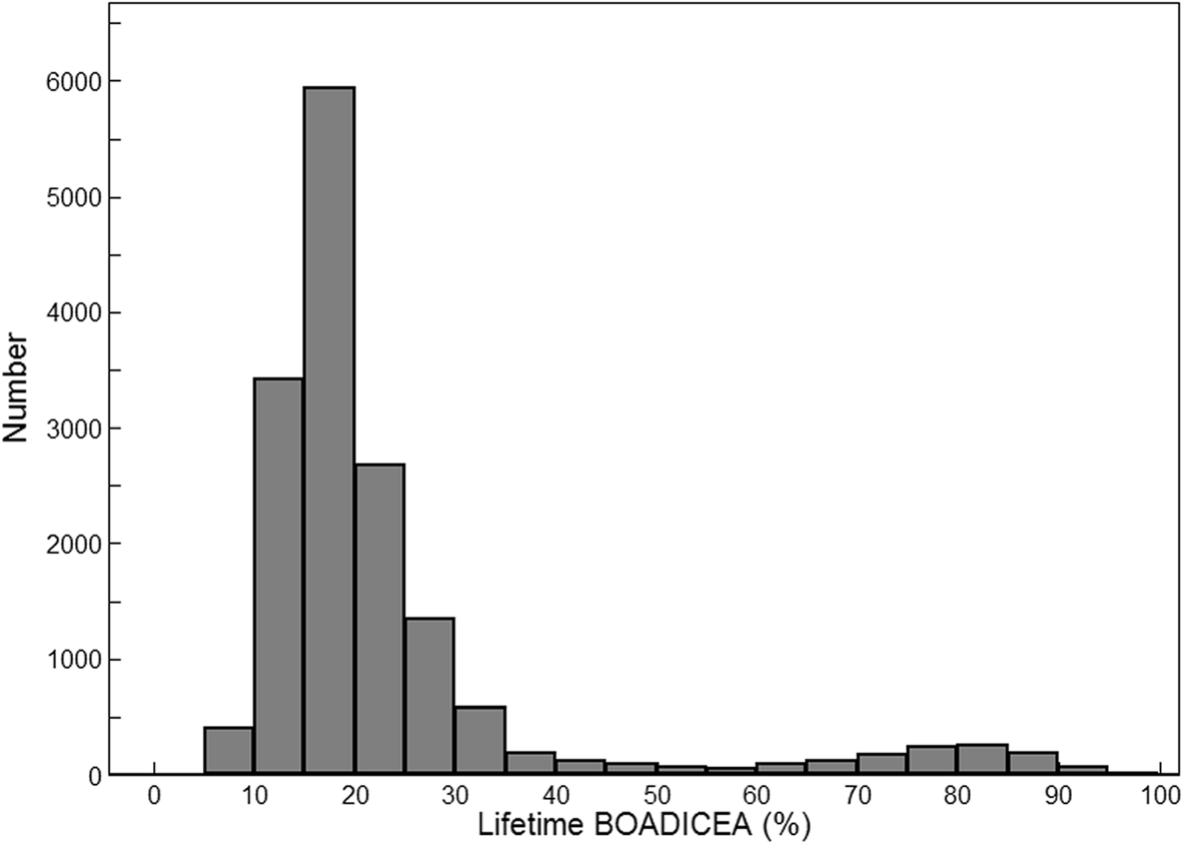
Distribution of lifetime risk from birth to age 80 years (as a percent) predicted from baseline pedigree data using the Breast and Ovarian Analysis of Disease Incidence and Carrier Estimation Algorithm (BOADICEA) for the cohort

Figure 1 shows the distribution of predicted lifetime breast cancer risk based on BOADICEA. The second peak starting at 60% lifetime risk is almost entirely due to identified *BRCA1* and *BRCA2* mutation carriers.

**Fig. 2:**
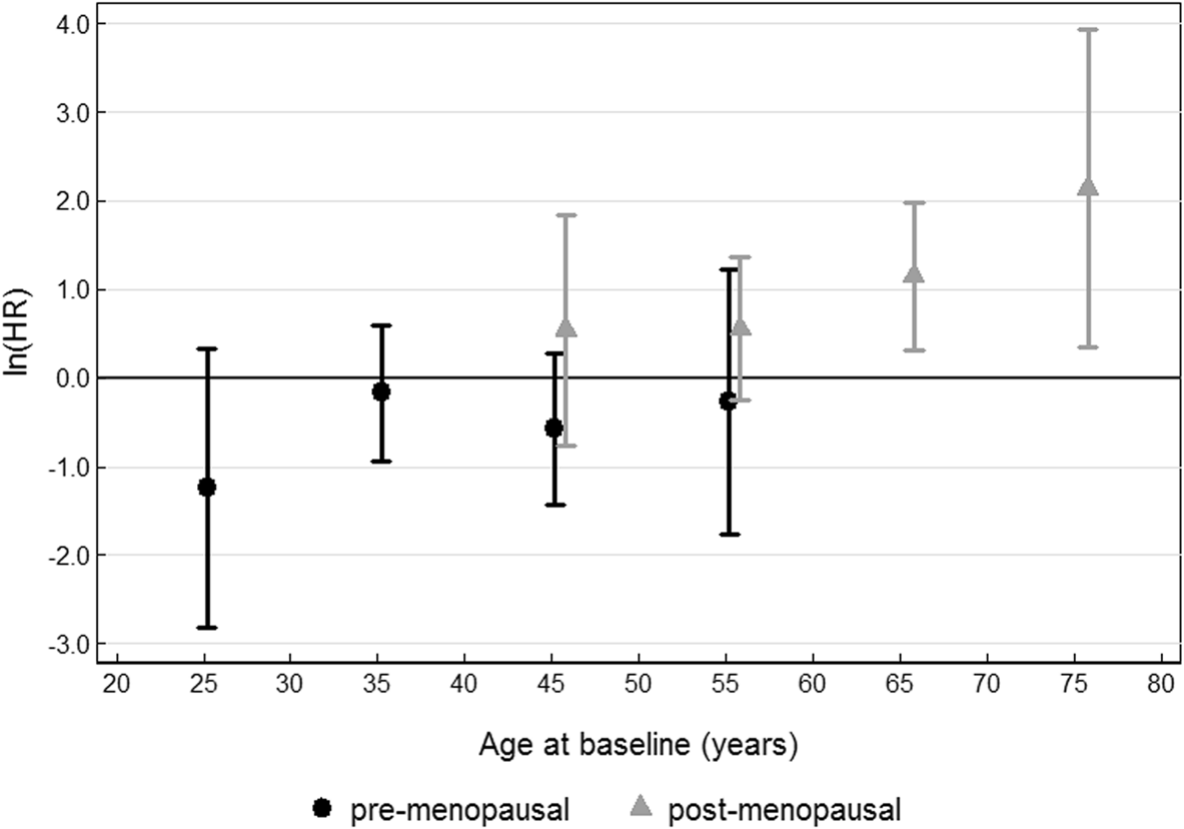
Estimated log hazard ratio, ln(HR), for log body mass index (per 5 kg/m^2^) for premenopausal and postmenopausal women as a function of age at baseline (in 10-year groups)

Figure 2 shows that the HR estimates for the association between greater BMI and breast cancer risk change from all being negative to all being positive when moving from age at baseline < 40 years to age > 60 years. The same changes in risk occur in moving from pre- to postmenopausal status.

The results from multivariable Cox models are shown in Tables 2 and 3. In Table 2, we show that the strength and direction of the BMI risk association depended on age at baseline (model II, HR = 1.05, *P* = 0.002) and on menopausal status (model III, HR = 3.68, *P* < 0.001) at baseline. When we modeled both together, the most important factor was baseline menopausal status because once this had been taken into account (HR = 2.91, *P* = 0.07), the BMI risk association with age at baseline (HR = 1.01, *P* = 0.6) was no longer significant. Comparison of log likelihoods from model IV versus model III shows that there was no evidence for an interaction between age at baseline and menopausal status (χ_1_^2^ = 0.01). A subsequent analysis found that there was also no evidence for an association with age at baseline for postmenopausal women (HR = 1.00, *P* = 1.0). Therefore, the best-fitting model for the BMI association included an interaction term between BMI and menopausal status only (model III).

**Table 2:** Adjusted hazard ratios (HRs) and 95% confidence intervals (CIs) from Cox proportional hazards modeling of body mass index, menopausal status and age at baseline

Model		HR^b^	95% CI	*P*	ΔLL^c^
I	Log body mass index^a^ (per 5 kg/m^2^)	1.28	0.91, 1.81	0.2	2.38
	Age at baseline, years	0.98	0.96, 1.00	0.1	
	Menopause, no/yes	1.12	0.88, 1.43	0.4	
II	Log body mass index^a^ (per 5 kg/m^2^)	0.13	0.03, 0.56	0.006	7.36
	Age at baseline, years	0.98	0.96, 1.00	0.1	
	Menopause, no/yes	1.12	0.88, 1.44	0.4	
	Log body mass index (per 5 kg/m^2^) × Age at baseline, years	1.05	1.02, 1.08	0.002	
III	Log body mass index^a^ (per 5 kg/m^2^)	0.68	0.41, 1.01	0.1	9.14
	Age at baseline, years	0.98	0.96, 1.00	0.1	
	Menopause, no/yes	1.12	0.87, 1.44	0.4	
	Log body mass index (per 5 kg/m^2^) × Menopause, no/yes	3.68	1.86, 7.28	< 0.001	
IV	Log body mass index^a^ (per 5 kg/m^2^)	0.40	0.06, 2.89	0.4	9.32
	Age at baseline, years	0.98	0.96, 1.00	0.1	
	Menopause, no/yes	1.12	0.88, 1.44	0.4	
	Log body mass index (per 5 kg/m^2^) × Age at baseline, years	1.01	0.97, 1.06	0.6	
	Log body mass index (per 5 kg/m^2^) × Menopause, no/yes	2.91	0.91, 9.31	0.07	

To account for clustering by family, robust 95% CIs are reported

*LL* log likelihood

^a^Adjusted for log baseline age as a quadratic

^b^Adjusted for history of benign breast disease, race/ethnicity, and education; stratified by year of birth (10-year groups) and study site

^c^Change in LL from the base model that includes benign breast disease, race/ethnicity, and education

**Table 3:** Adjusted hazard ratios (HRs) and 95% confidence intervals (CIs) from Cox proportional hazards modelling of body mass index, menopausal status, age, and BOADICEA 1-year risk of breast cancer at baseline

Model		HR^b^	95% CI	*P*	ΔLL^c^
V	Log body mass index^a^ (per 5 kg/m^2^)	0.75	0.46, 1.22	0.2	158.03
	Log 1-year BOADICEA^a^ (%)	2.05	1.89, 2.23	< 0.001	
	Age at baseline, years	0.98	0.96, 1.00	0.1	
	Menopause, no/yes	1.04	0.81, 1.32	0.8	
	Log body mass index (per 5 kg/m^2^) × Menopause, no/yes	3.36	1.71, 6.62	< 0.001	
VI	Log body mass index^a^ (per 5 kg/m^2^)	0.75	0.46, 1.22	0.2	158.03
	Log 1-year BOADICEA^a^ (%)	2.05	1.86, 2.26	< 0.001	
	Age at baseline, years	0.98	0.96, 1.00	0.1	
	Menopause, no/yes	1.03	0.80, 1.34	0.8	
	Log body mass index (per 5 kg/m^2^) × Menopause, no/yes	3.36	1.71, 6.63	< 0.001	
	Menopause, no/yes × Log 1-year BOADICEA^a^ (%)	1.00	0.85, 1.19	1.0	
VII	Log body mass index^a^ (per 5 kg/m^2^)	0.70	0.41, 1.18	0.2	158.24
	Log 1-year BOADICEA^a^ (%)	2.06	0.89, 2.24	< 0.001	
	Age at baseline, years	0.98	0.96, 1.00	0.1	
	Menopause, no/yes	1.04	0.81, 1.32	0.8	
	Log body mass index (per 5 kg/m^2^) × Menopause, no/yes	3.46	1.75, 6.84	< 0.001	
	Log body mass index^a^ (per 5 kg/m^2^) × log 1-year BOADICEA^a^ (%)	1.13	0.79, 1.62	0.5	
VIII	Log body mass index^a^ (per 5 kg/m^2^)	0.66	0.38, 1.14	0.1	158.54
	Log 1-year BOADICEA^a^ (%)	2.07	1.90, 2.25	< 0.001	
	Age at baseline, years	0.98	0.96, 1.00	0.1	
	Menopause, no/yes	1.04	0.82, 1.32	0.8	
	Log body mass index (per 5 kg/m^2^) × Menopause, no/yes	3.92	1.86, 8.28	< 0.001	
	Log body mass index^a^ (per 5 kg/m^2^) × Log 1-year BOADICEA^a^ (%)	1.27	0.81, 2.00	0.3	
	Menopause, no/yes × Log body mass index^a^ (per 5 kg/m^2^) × Log 1-year BOADICEA^a^ (%)	0.73	0.34, 1.57	0.4	

To account for clustering by family, robust 95% CIs are reported

*LL* log likelihood, *BOADICEA* Breast and Ovarian Analysis of Disease Incidence and Carrier Estimation Algorithm

^a^Adjusted for log baseline age as a quadratic

^b^Adjusted for history of benign breast disease, race/ethnicity, and education; stratified by year of birth (10-year groups) and study site

^c^Change in LL from the base model that includes history of benign breast disease, race/ethnicity, and education

Table 3 shows the model fits after taking familial risk into account. The most parsimonious best fitting model was model V, which shows that after the BMI association was fitted as a function of menopausal status (HR = 3.36, *P* < 0.001), there was evidence of an association with familial risk (as represented by the log 1-year BOADICEA estimate in model V; HR = 2.05, *P* < 0.001). The other models show that there was no evidence for an interaction of 1-year BOADICEA score with menopausal status (model VI, HR = 1.00, *P* = 1.0), with BMI (model VII, HR = 1.13, *P* = 0.5), or with the interaction of BMI and menopausal status (model VIII, HR = 0.73, *P* = 0.4). That is, there was no evidence that the multiplicative interaction between BMI and menopausal status differed by familial risk irrespective of how we modeled the BMI association We also re-analyzed the data by including women with missing menopausal status and putting them in a category of their own. This made no difference to our general findings of no evidence for gene–environment interactions on the multiplicative scale.

**Fig. 3:**
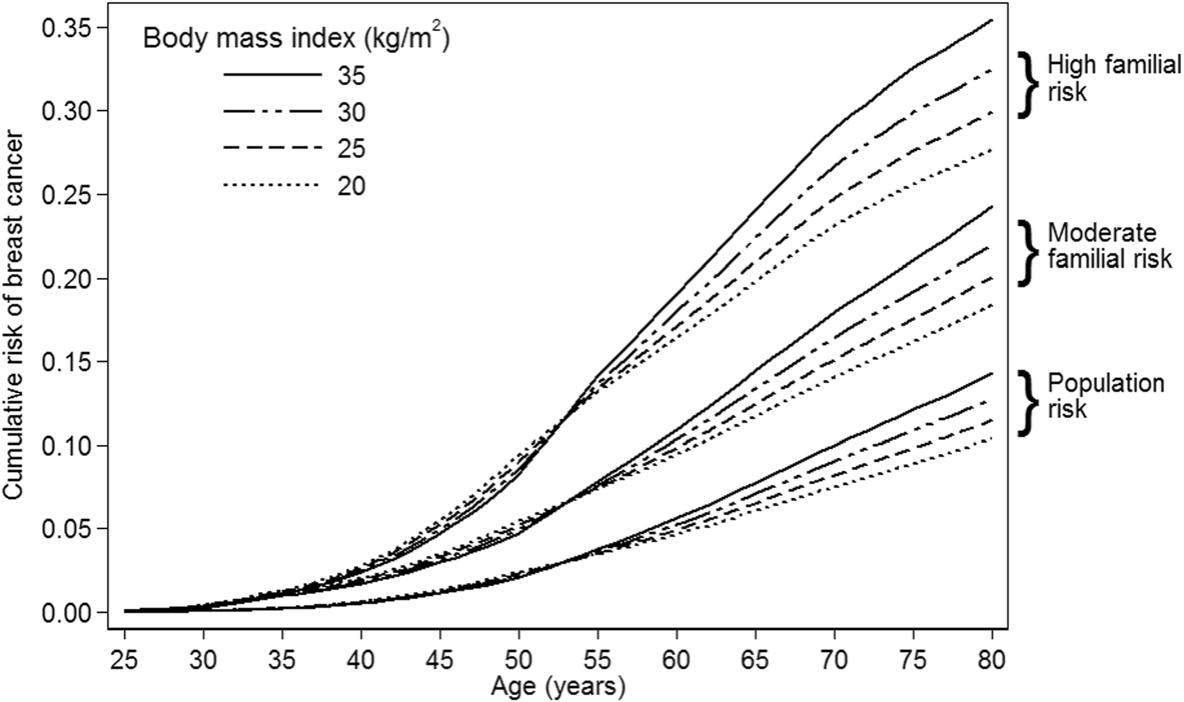
Predicted age-specific cumulative risk (from birth) of breast cancer, based on model V (see Table 3), by body mass index and familial risk at baseline, where moderate familial risk is equivalent to having one affected first-degree relative and high familial risk is equivalent to having two affected first-degree relatives

Figure 3 shows the overall implications of the study estimates on the predicted age-specific cumulative risk for women with different baseline BMI and familial risk and age 50 years at menopause. In terms of absolute risk, the risk difference for premenopausal women is small when comparing those in the lowest BMI category with those in the highest BMI category. In contrast, the corresponding risk difference for postmenopausal women is much larger and in the opposite direction. The latter difference in absolute risk is even more so for women with a greater familial risk (e.g. for cumulative risk to age 80 years, 8% for women with high familial risk versus 4% for population risk).

## Discussion

Using a large international prospective cohort enriched for women with a family history of breast cancer [28] we have found that the *absolute* breast cancer risk gradient with BMI increases with age after menopause, and with underlying familial risk. There are three key findings of clinical and biological significance and they are illustrated in Fig. 3.

First, we found that greater BMI at a young adult age is associated with a decreased risk of breast cancer, as have others [44]. We have shown that this negative association with BMI does not translate into a substantial influence on absolute risk of breast cancer.

Second, our modeling confirms that BMI is associated with an increase in risk once a woman becomes postmenopausal. In terms of differences in absolute risk, it is not until a woman is in her mid to late 50s that the risk manifests; the influence on absolute risk then increases with age.

Third, our modeling predicts that the greater a woman’s familial risk, the greater the influence of BMI on her absolute postmenopausal breast cancer risk. We base this on our finding that, in terms of multiplicative risk, the association of breast cancer with BMI did not differ for women at different underlying familial risk (Table 3). Unlike most other cohorts, our enriched cohort has adequate statistical power to examine interactions with underlying familial risk [28, 45, 46]. We also created a continuous measure of familial risk using multi-generational pedigree information and the BOADICEA model to estimate 1-year and lifetime (from birth) risk of breast cancer [35, 36, 43].

As illustrated in Fig. 1, about one third of our cohort has a lifetime risk above the clinically relevant cutoff of 20% [47, 48]. Figure 3 shows that our observed lack of multiplicative interaction means that the difference in absolute risk between women at higher compared with lower BMI is greater for those women who are at higher underlying familial risk. Our finding of a lack of multiplicative interaction between BMI and 1-year BOADICEA score is consistent with the lack of multiplicative interactions with BMI and more than 100 genetic variants found by a large pooled case–control analysis of almost 60,000 women [49].

One potential limitation is that a change in the BMI association with baseline menopausal status could be due to unmeasured confounders. For this to happen, such confounders would have to have a similar menopausal-dependent risk association. As most risk factors related to BMI (e.g., physical activity) do not have clear differences in association by menopausal status, unmeasured confounding is not likely. Other limitations include limited power to address issues specific to mutation carriers and hormone receptor status of tumors. The evidence of whether the protective association of BMI in early life applies solely to estrogen receptor negative disease is less consistent, with contradictory findings from two meta-analyses [50, 51] and a recent study based on a pooled study [52].

Although the negative breast cancer risk association with childhood and adolescent BMI is small in terms of absolute risk, understanding this in the light of the genome-wide association studies results [18, 19], which also support a negative association of BMI with breast cancer risk at a young age, could aid in understanding the role of breast development in breast cancer susceptibility. While increased glucose and other nutrients might alter the ability of *BRCA1* to function as a tumor suppressor [53], pre-pubertal estrogen exposure could increase the ability of major breast cancer susceptibility genes to prevent breast cancer through cellular differentiation [54].

An explanation of the negative association with childhood and adolescent BMI might be found in the growth of mammographically dense tissue and changes to the architecture surrounding the mammographically dense tissue, which develops and grows rapidly in adolescence [55]. Importantly, as we show here, although higher BMI is associated with reduced breast cancer risk before menopause, the direction of the association is reversed post-menopause and is of far more consequence in terms of absolute risk.

Laboratory studies have given insights into the mechanisms that might explain why weight gain and metabolically rich environments increase postmenopausal breast cancer risk, with implications for prevention [56]. These mechanisms include conversion of androgens to estrogens in adipose tissue [57], but could also include inflammation and metabolic processes related to cancer risk [58, 59] and changes to epigenetically regulated genes such as *BRCA1* [53].

## Conclusions

In summary, the negative association with BMI in premenopausal women has a much smaller influence on absolute risk than the positive association with BMI in postmenopausal women. Women at higher familial risk have a much larger difference in absolute risk depending on their BMI than women at lower familial risk.

Our modeling predicted that, for young and premenopausal women, the decrease in breast cancer risk associated with increasing BMI does not have a substantial influence on absolute risk in those periods of life. Our modeling also predicted that the *absolute* breast cancer risk gradient with BMI increases with age post menopause, and with underlying familial risk. We argue, therefore, that there is no discrepancy between the conclusions of the Mendelian randomization studies [18, 19] and the epidemiological literature (see “Background”). The genetically driven protective role of BMI on breast cancer risk in early adulthood appears to be of little consequence in terms of absolute risk and is overtaken by an environmentally driven deleterious role of greater adult BMI in later life that is even more important for women at increased genetic risk. Given that age-adjusted BMI is correlated between early and mid-adulthood [60], maintaining a healthy weight throughout adult life is of clinical significance for all women, and especially those with a family history of breast cancer.

## Abbreviations

BCFR: Breast Cancer Family Registry
BMI: Body mass index
BOADICEA: Breast and Ovarian Analysis of Disease Incidence and Carrier Estimation Algorithm
kConFab: Kathleen Cuningham Foundation Consortium for Research into Familial Breast Cancer
Prof-SC: Breast Cancer Prospective Family Study Cohort
SNP: Single-nucleotide polymorphism
